# Plasma p-tau181 Level Predicts Neurodegeneration and Progression to Alzheimer's Dementia: A Longitudinal Study

**DOI:** 10.3389/fneur.2021.695696

**Published:** 2021-09-07

**Authors:** Yan-Li Wang, Jinglong Chen, Zhong-Li Du, Haoyi Weng, Yuan Zhang, Runzhi Li, Ziyan Jia, Mengfan Sun, Jiwei Jiang, Fang-Ze Wang, Jun Xu, Alzheimer's Disease Neuroimaging Initiative

**Affiliations:** ^1^Department of Neurology, Beijing Tiantan Hospital, Capital Medical University, Beijing, China; ^2^Department of Geriatric Medicine, China National Clinical Key Specialty, Guangzhou First People's Hospital, School of Medicine, South China University of Technology, Guangzhou, China; ^3^National Center for Clinical Laboratories, Beijing Hospital, National Center of Gerontology, Beijing, China; ^4^Institute of Geriatric Medicine, Chinese Academy of Medical Sciences, Beijing, China; ^5^Shenzhen WeGene Clinical Laboratory, Shenzhen, China; ^6^WeGene, Shenzhen Zaozhidao Technology Co. Ltd., Shenzhen, China; ^7^Hunan Provincial Key Lab on Bioinformatics, School of Science and Engineering, Central South University, Shenzhen, China; ^8^Department of Cardiology, Weifang People's Hospital, Weifang, China

**Keywords:** Alzheimer's disease, plasma p-tau181, cognition, cerebrospinal fluid (CSF), NeuroImage

## Abstract

**Background:** Plasma-based biomarkers would be potential biomarkers for early diagnosis of Alzheimer's disease (AD) because they are more available and cost-effective than cerebrospinal fluid (CSF) or neuroimaging. Therefore, we aimed to evaluate whether phosphorylated tau181 (p-tau181) in plasma could be an accurate AD predictor.

**Methods:** Participants from the ADNI database included 185 cognitively unimpaired subjects with negative Aβ (CU–), 66 subjects with pre-clinical AD (CU with positive Aβ), 164 subjects with mild cognitive impairment with negative Aβ (MCI–), 254 subjects with prodromal AD (MCI with positive Aβ), and 98 subjects with dementia. Multiple linear regression models, linear mixed-effects models, and local regression were used to explore cross-sectional and longitudinal associations of plasma p-tau181 with cognition, neuroimaging, or CSF biomarkers adjusted for age, sex, education, and APOE genotype. Besides, Kaplan–Meier and adjusted Cox-regression model were performed to predict the risk of progression to dementia. Receiver operating characteristic analyses were performed to evaluate the predictive value of p-tau181.

**Results:** Plasma p-tau181 level was highest in AD dementia, followed by prodromal AD and pre-clinical AD. In pre-clinical AD, plasma p-tau181 was negatively associated with hippocampal volume (β = −0.031, *p*-value = 0.017). In prodromal AD, plasma p-tau181 was associated with decreased global cognition, executive function, memory, language, and visuospatial functioning (β range −0.119 to −0.273, *p*-value < 0.05) and correlated with hippocampal volume (β = −0.028, *p*-value < 0.005) and white matter hyperintensity volume (WMH) volume (β = 0.02, *p*-value = 0.01). In AD dementia, increased plasma p-tau181 was associated with worse memory. In the whole group, baseline plasma p-tau181 was significantly associated with longitudinal increases in multiple neuropsychological test *z*-scores and correlated with AD-related CSF biomarkers and hippocampal volume (*p*-value < 0.05). Meanwhile, CU or MCI with high plasma p-tau181 carried a higher risk of progression to dementia. The area under the curve (AUC) of the adjusted model (age, sex, education, APOE genotype, and plasma p-tau181) was 0.78; that of additionally included CSF biomarkers was 0.84.

**Conclusions:** Plasma p-tau181 level is related to multiple AD-associated cognitive domains and AD-related CSF biomarkers at the clinical stages of AD. Moreover, plasma p-tau181 level is related to the change rates of cognitive decline and hippocampal atrophy. Thus, this study confirms the utility of plasma p-tau181 as a non-invasive biomarker for early detection and prediction of AD.

## Introduction

Alzheimer's disease (AD) neuropathologically characterized by amyloid beta (Aβ) and tau, the major components of senile plaques and neurofibrillary tangles, respectively, is the most common neurodegenerative disorder of dementia causing progressive cognitive decline (1). CSF Aβ42, total tau (t-tau), and phosphorylated tau (p-tau) were core AD-diagnostic biomarkers and CSF Aβ42/Aβ 40 could further improve the diagnostic accuracy (2–4).

The diagnostic criteria for AD was established based on amyloidosis, tau pathology, and neurodegeneration derived from cerebrospinal fluid (CSF), positron emission tomography (PET), and magnetic resonance imaging (MRI) proposed by the National Institute of Aging-Alzheimer Association (5, 6). Though PET and CSF biomarkers are invaluable in AD-related brain pathology, the use of PET imaging and lumbar puncture is restricted to limited centers because of high prices, radiopharmaceuticals, and invasiveness. Conventional structural MRI neuroimaging is often used to evaluate AD progression. However, the advanced MR techniques are of limited value in AD diagnosis due to high heterogeneity, low sensitivity, and low signal-to-noise ratios (7).

Therefore, an affordable, non-invasive means is essential for large-scale screening programs and longitudinal studies. The previous study has developed a new ATN framework, focused on plasma biomarkers including Aβ_42_/Aβ_40_ ratio (A), plasma p-tau181 (T), and neurofilament light (N) (8). Plasma Aβ_42_/Aβ_40_ ratio was decreased in cognitively normal subjects with subjective cognitive decline, the earliest stage of AD, and plasma Aβ_42_/Aβ_40_ level can predict amyloid-PET status (9, 10). Nevertheless, peripheral production of Aβ peptides makes it difficult for clinical practice (11). Plasma neurofilament light level was increased in patients with AD (12, 13), but not specific for AD given that many other neurodegenerative diseases such as amyotrophic lateral sclerosis, Creutzfeldt–Jakob disease, and frontotemporal dementia showed significantly high values (14–16). Recent prospective studies found that blood p-tau181 can be a potential diagnostic marker of AD (17–19). A study of familial AD indicated that plasma p-tau181 level was increased before symptom onset (20). However, longitudinal analysis is scarce on plasma p-tau181 prognostic value for progression to dementia and relationships between plasma p-tau181 and multiple AD-associated cognitive domains remain unclear.

In the present study, we investigated longitudinal associations of plasma p-tau181 with AD-related CSF biomarkers, multiple cognitive domains, and volumes of hippocampus and white matter hyperintensity (WMH). Moreover, we assessed the value of plasma p-tau181 in predicting progression to dementia and compared the diagnostic values of different models.

## Methods

### Study Population

Participants were selected from the Alzheimer's Disease Neuroimaging Initiative (ADNI) data (http://adni.loni.usc.edu) in October 2020. The ADNI database was initiated in 2003 to detect MCI and early AD dementia progression based on the combinations of CSF or plasma biomarkers, serial MRI, PET, and clinical and neuropsychological information. This project was approved by institutional review boards of all participating institutions and written informed consent was obtained from all participants before inclusion in the study.

The study included 767 ADNI participants with baseline plasma p-tau181. Definitions of the participant classifications have been described in the previous study, including cognitively unimpaired (CU), mild cognitive impairment (MCI), and dementia (21). Besides, in this study, the threshold values of Aβ were 880 pg/ml for CSF Aβ1-42 or 1.11 SUVR for AV45-PET scan (22). Then, CU and MCI subjects were split into four subgroups: subjects with negative Aβ-related pathological changes (CU– and MCI–) and subjects with positive Aβ-related pathological changes (CU+ and MCI+). CU+ subjects were regarded as patients with pre-clinical AD and MCI+ subjects were regarded as patients with prodromal AD. The enrollment flow chart is given in Supplementary Figure 1.

### Plasma p-tau181 Quantification

Level of plasma p-tau181 was quantified with the Single Molecule array (Simoa) technique combined with two monoclonal antibodies (Tau12 and AT270), as described previously (17). Moreover, the analysis of plasma p-tau181 was carried out at the Clinical Neurochemistry Laboratory, University of Gothenburg, Sweden.

### Cognitive Evaluation

The general cognition was assessed by Mini-Mental State Examination (MMSE) and Montreal Cognitive Assessment (MoCA), and composite scores were used to reflect memory, executive function, and language-related and visuospatial domains. All these neuropsychological tests were evaluated at baseline and yearly thereafter.

### Neuroimaging Analyses

The average of the mean florbetapir standard uptake value ratio (SUVR) was calculated using four cortical regions (frontal, anterior cingulate, precuneus, and parietal cortex) normalized to the whole cerebellum. WMH volume was computed with the segmentation of high-resolution 3D T1-weighted and FLAIR sequences based on a Bayesian approach. The average volume of the right and left hippocampus was obtained using FreeSurfer software (http://surfer.nmr.mgh.harvard.edu/fswiki).

### CSF Measurements

Levels of Aβ, total tau (t-tau), and p-tau in CSF were measured using Elecsys immunoassays at the Clinical Neurochemistry Laboratory, University of Gothenburg, Sweden (BioFINDER) or at the Biomarker Research Laboratory, University of Pennsylvania, USA (23).

### Statistical Analyses

The level of plasma p-tau181 was compared between five subgroups with the Mann–Whitney *U* test. The rate of change in plasma p-tau181 was calculated in linear mixed-effects models with age, sex, education, and APOE genotype as covariates. Baseline associations of plasma p-tau181 with cognition and other biomarkers were tested by linear regression models adjusting for age, sex, education, and APOE genotype. Associations of baseline plasma p-tau181 with longitudinal level of other biomarkers were tested by linear mixed-effects models. The local regression was employed for associations between baseline plasma p-tau181 and the change rate of other biomarkers.

Furthermore, clinical progression to dementia was estimated using Kaplan–Meier survival curves with log-rank test. The receiver operating characteristic (ROC) curves and the area under the curve (AUC) of the key biomarkers of plasma and CSF were calculated to predict progression to dementia. All tests used a significance level of *p* < 0.05 and all statistical analyses were performed with R (version 1.1.383) and GraphPad (Prism 8.0.2).

## Results

### Demographic Data

**Figure 1:**
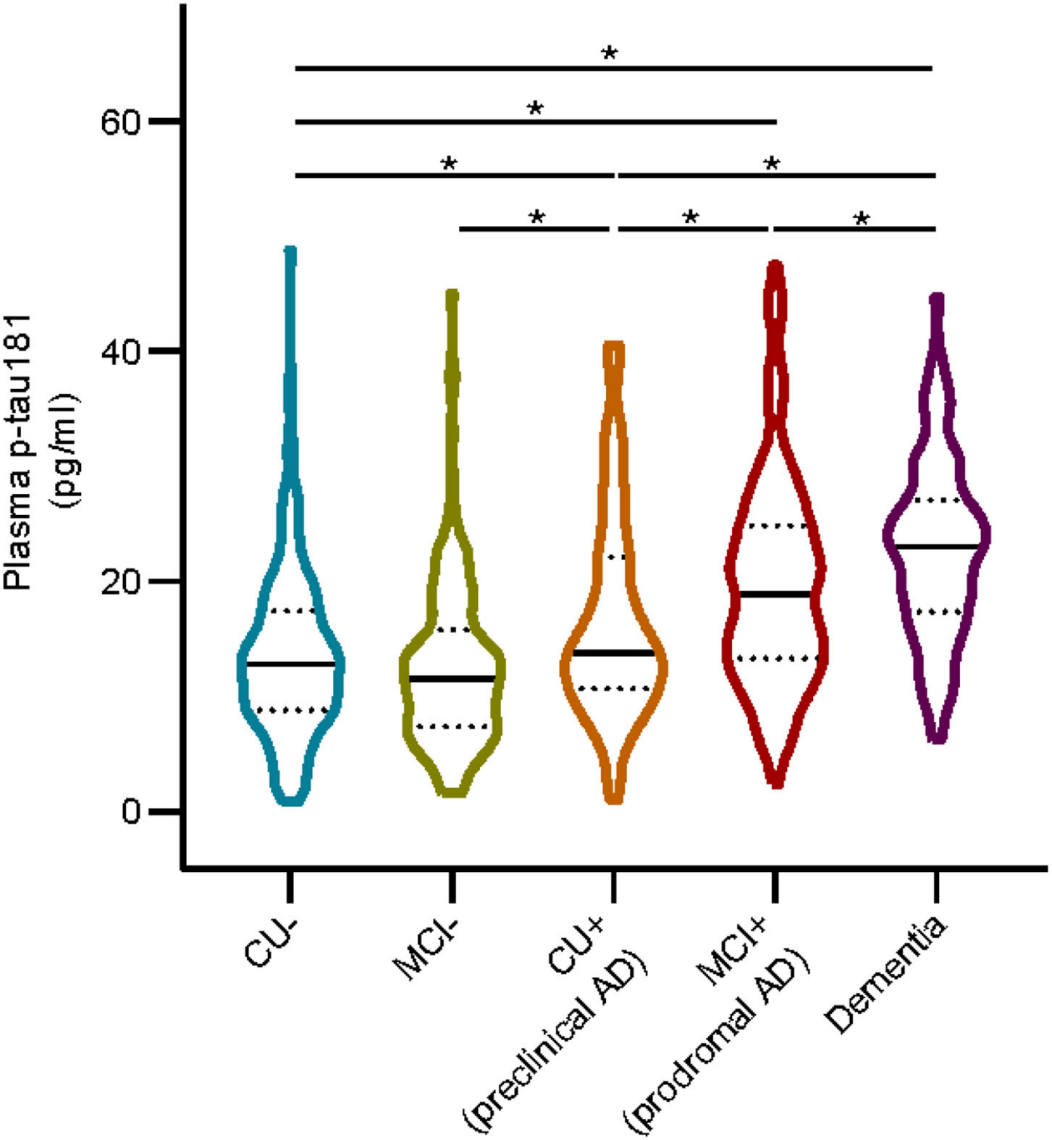
Plasma p-tau 181 in different groups. Asterisk indicates a significant difference between groups (*p*-value < 0.05, Mann–Whitney *U* test). CU–, cognitively unimpaired with negative Aβ; MCI–, mild cognitive impairment with negative Aβ; CU+, cognitively unimpaired with positive Aβ; MCI+, mild cognitive impairment with positive Aβ.

We included 767 subjects (mean ± SD age, 72.3 ± 7.1 years; education, 16.3 ± 2.6 years), of whom 366 were women (47.7%). Other descriptive statistics of the subjects are given in Table 1. The cohort were divided into five subgroups: 185 from the CU– group (cognitively unimpaired subjects with negative Aβ), 164 from the MCI– group (MCI– subjects with negative Aβ), 66 from the CU+ group (pre-clinical AD, CU– subjects with positive Aβ), 254 from the MCI+ group (prodromal AD, MCI– subjects with positive Aβ), and 98 from the dementia group. Figure 1 shows that the level of plasma p-tau181 is highest in dementia, followed by that in prodromal AD and pre-clinical AD and the lowest level were found in MCI– (*p* < 0.05), and there is no significant difference between MCI– and CU–.

**Table 1:** Demographic and clinical characteristics of participants.

	**CU–**	**MCI–**	**CU+**	**MCI+**	**Dementia**
			**(Preclinical AD)**	**(Prodromal AD)**	
***N***	185	164	66	254	98
Age, mean ± SD, years	72.34 ± 5.99	69.67 ± 7.63	74.17 ± 6.32	72.57 ± 6.99	74.70 ± 7.74
Female, *n* (%)	98 (53)	79 (48.2)	38 (57.6)	113 (44.5)	38 (38.77)
Education, mean ± SD, years	16.79 ± 2.58	16.28 ± 2.44	16.12 ± 2.44	16.28 ± 2.71	15.69 ± 2.64
APOE ε4 carriers, *n* (%)	43 (23.20)	36 (22)	30 (45.50)	165 (65)	69 (70.41)
Cognitive scores, mean ± SD					
MMSE	29.06 ± 1.23	28.55 ± 1.44	28.94 ± 1.24	27.8 ± 1.77	23.07 ± 2.03
MOCA	25.93 ± 2.33	24.03 ± 3.14	25.35 ± 2.29	23.11 ± 3.07	17.41 ± 4.44
ADNI_EF	0.96 ± 0.83	0.63 ± 0.78	0.69 ± 0.81	0.27 ± 0.91	−0.84 ± 0.92
ADNI_Mem	1.09 ± 0.56	0.65 ± 0.64	0.99 ± 0.66	0.20 ± 0.65	−0.90 ± 0.50
ADNI_Lan	0.84 ± 0.66	0.53 ± 0.72	0.85 ± 0.72	0.28 ± 0.74	−0.71 ± 0.96
ADNI_VS	0.22 ± 0.60	0.10 ± 0.68	0.17 ± 0.57	−0.05 ± 0.73	−0.47 ± 0.94
Neuroimaging, mean ± SD					
WMH volume, cm^3^	4.66 ± 6.11	5.21 ± 7.03	10.07 ± 17.66	8.12 ± 9.88	8.45 ± 8.85
AV45 PET, SUVR	1.06 ± 0.14	1.01 ± 0.05	1.26 ± 0.19	1.35 ± 0.19	1.41 ± 0.20
Hippocampal volume, cm^3^	6.71 ± 0.78	6.58 ± 0.92	6.67 ± 0.72	6.26 ± 0.87	5.56 ± 0.84
Plasma biomarker, mean ± SD (pg/ml)					
P-tau 181	13.64 ± 7.33	12.58 ± 7.08	16.56 ± 8.94	19.77 ± 9.03	22.80 ± 7.71
CSF biomarkers, mean ± SD (pg/ml)					
Aβ1-42	1,560.65 ± 610.72	1,607.33 ± 512.28	904.41 ± 549.16	770.03 ± 308.75	694.78 ± 474.11
T-tau	224.47 ± 78.97	212.62 ± 75.88	257.80 ± 114.81	315.08 ± 139.71	383.46 ± 157.63
P-tau	20.15 ± 7.83	18.60 ± 7.15	24.97 ± 11.94	31.27 ± 15.54	38.03 ± 16.16
T-tau/Aβ1–42	0.17 ± 0.11	0.14 ± 0.05	0.35 ± 0.2	0.46 ± 0.26	0.67 ± 0.37
P-tau/Aβ1–42	0.02 ± 0.01	0.01 ± 0	0.03 ± 0.02	0.05 ± 0.03	0.07 ± 0.04

*CU, cognitively unimpaired; MCI, mild cognitive impairment; –, with negative Aβ; +, with positive Aβ; MMSE, mini-mental state examination; MOCA, Montreal cognitive assessment; ADNI_EF, executive domain composite score; ADNI_Mem, memory domain composite score; ADNI_Lan, language-related composite score; ADNI_VS, visuospatial domain composite score; WMH, white matter hyperintensity; FDG-PET, F18-fluorodeoxyglucose positron emission tomography; AV45 PET, florbetapir PET (amyloid protein imaging); SUVR, standardized uptake value ratio; P-tau, phosphorylated tau protein; Aβ, amyloid-β; T-tau, total tau protein*.

### Cross-sectional Associations of Plasma p-tau181 With CSF Biomarkers, Cognition, and Imaging Markers

**Figure 2:**
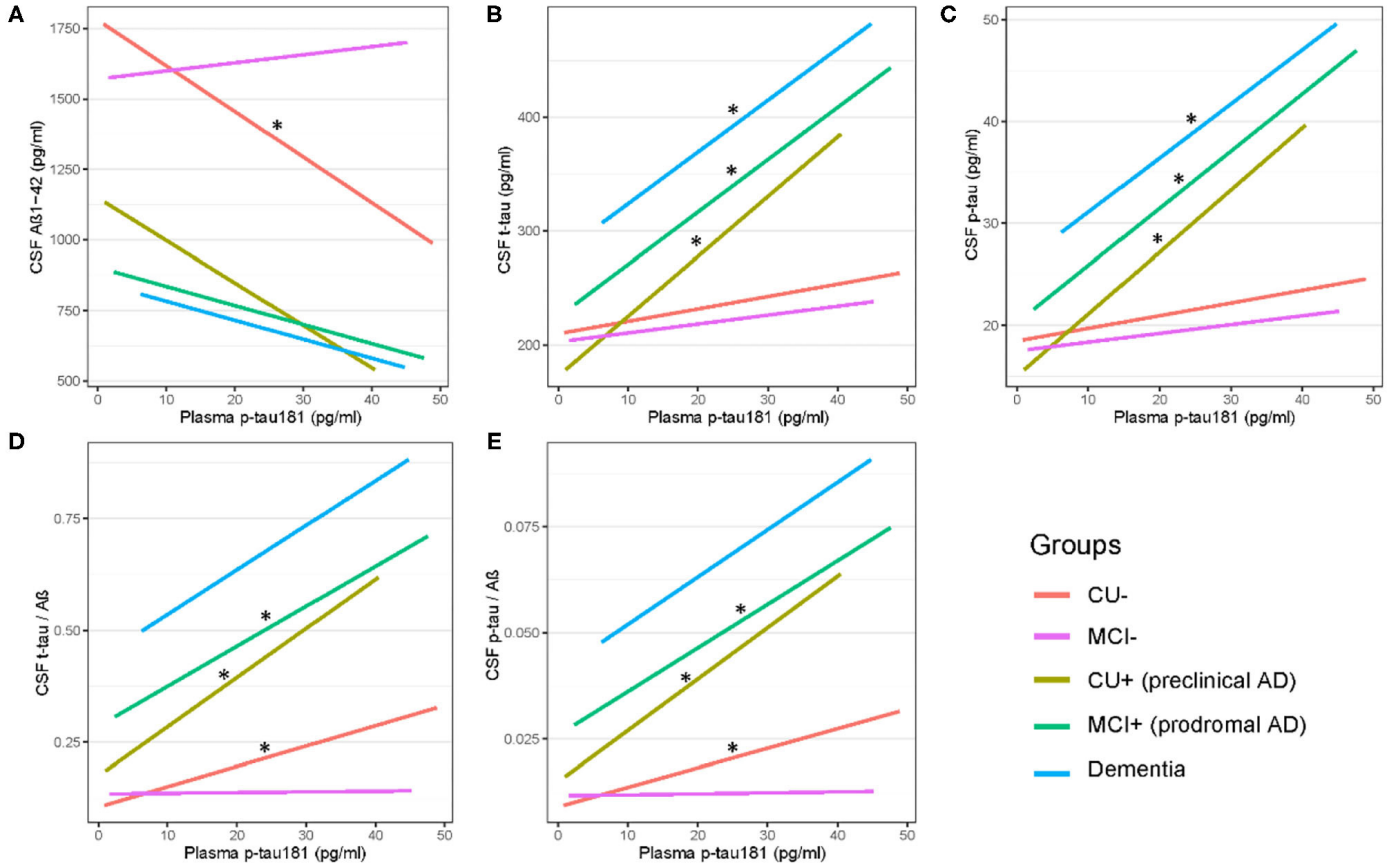
**(A–E)** Cross-sectional associations of plasma p-tau 181 with CSF biomarkers across AD dementia staging. Significant correlations are indicated by “*” (*p* < 0.05). CU–, cognitively unimpaired with negative Aβ; MCI–, mild cognitive impairment with negative Aβ; CU+, cognitively unimpaired with positive Aβ; MCI+, mild cognitive impairment with positive Aβ.

**Figure 3:**
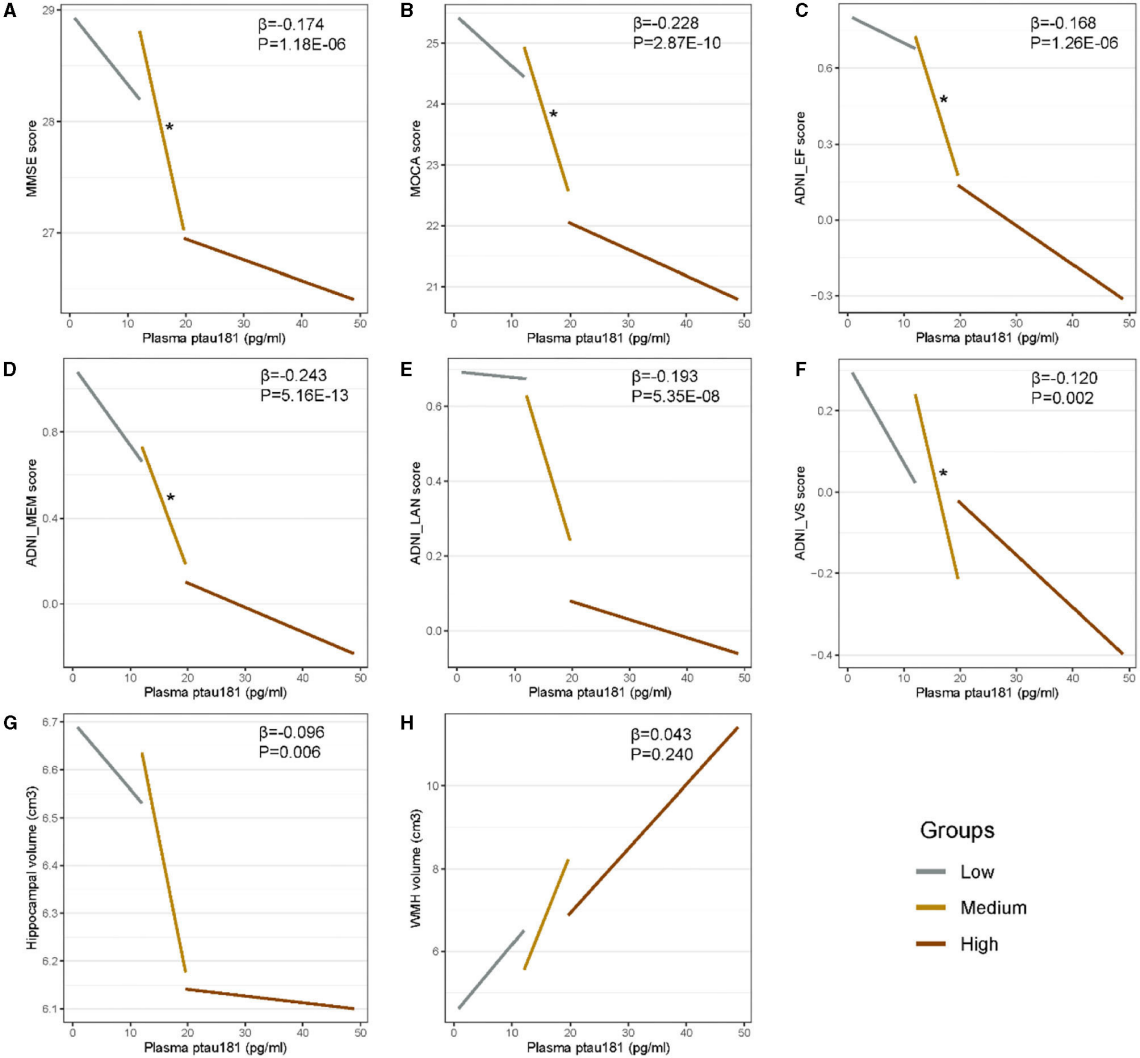
**(A–H)** Cross-sectional associations of plasma p-tau181 with cognition and neuroimaging biomarkers. Plasma p-tau181 is categorized into three 33% terciles: low < 12.03 pg/ml, medium 12.04–19.63 pg/ml, and high > 19.65 pg/ml. Significant correlations (*p* < 0.05) are indicated by “*” in different levels of plasma p-tau181.

Figure 2 shows that there are significant associations of plasma p-tau181 with CSF t-tau (pre-clinical AD: β = 0.46, *p* = 0.001; prodromal AD: β = 0.23, *p* = 0.0003), CSF p-tau (β = 0.51, *p* = 0.0003; β = 0.26, *p* = 3.29E−05), CSF t-tau/Aβ (β = 0.43, *p* = 0.003; β = 0.21, *p* = 0.0008), and CSF p-tau/Aβ (β = 0.45, *p* = 0.002; β = 0.23, *p* = 0.0002) in both pre-clinical AD and prodromal AD. Besides, significant associations of plasma p-tau181 with CSF Aβ (β = −0.17, *p* = 0.037), CSF t-tau/Aβ (β = 0.25, *p* = 0.0009), and CSF p-tau/Aβ (β = 0.24, *p* = 0.001) were found in the CU– group, and significant associations of plasma p-tau181 with CSF t-tau (β = 0.22, *p* = 0.037) and CSF p-tau (β = 0.25, *p* = 0.016) were found in the dementia group. As for the cohort, plasma p-tau181 was significantly associated with CSF Aβ and above biomarkers (Supplementary Table 1). Figure 3 indicates that there may be non-linear relationship between plasma p-tau181 and cognition. In the plasma p-tau181 medium subgroup (12.04–19.63 pg/ml), there were significant associations between plasma p-tau181 and MMSE (β = −0.22, *p* = 0.0003), MOCA (β = −0.17, *p* = 0.005), ADNI_EF (β = −0.12, *p* = 0.038), ADNI_Mem (β = −0.17, *p* = 0.003), and ADNI_VS (β = −0.18, *p* = 0.005). Additionally, plasma p-tau181 was also correlated with ADNI_Lan (β = −0.12, *p* = 0.002) and hippocampal volume (β = −0.096, *p* = 0.006) in the total study population (Supplementary Table 2). There was no significant associations between plasma p-tau181 and white matter hyperintensity (WMH) volume at baseline.

### Longitudinal Associations of Plasma p-tau181 With Cognition and Imaging Markers

**Figure 4:**
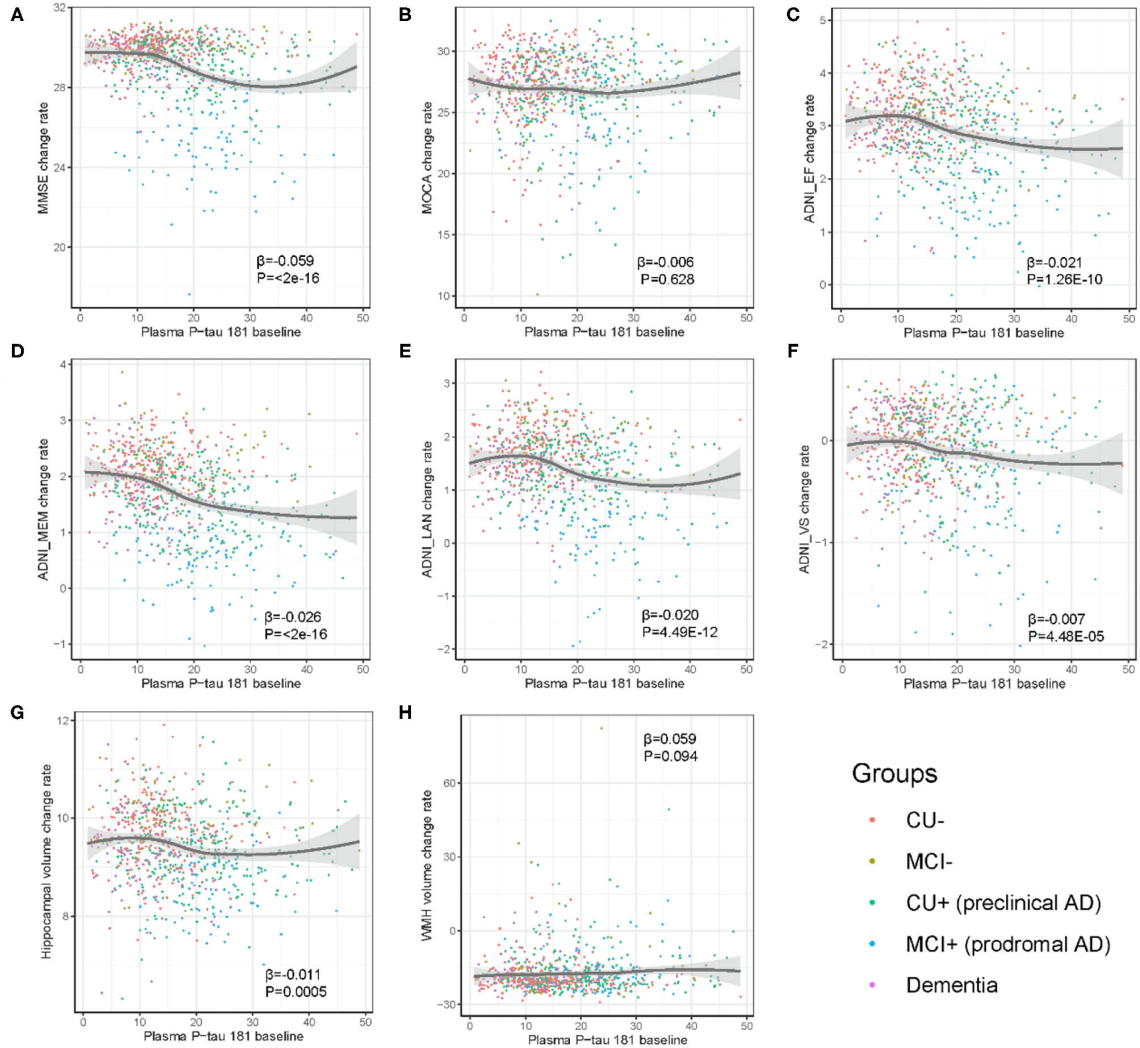
**(A–H)** Association of baseline level of plasma p-tau181 with yearly change of cognition and neuroimaging biomarkers. The rate of change in biomarkers was calculated in linear mixed effects model, adjusted for age, sex, education, and APOE genotype. CU–, cognitively unimpaired with negative Aβ; MCI–, mild cognitive impairment with negative Aβ; CU+, cognitively unimpaired with positive Aβ; MCI+, mild cognitive impairment with positive Aβ.

In prodromal AD, plasma p-tau181 was significantly correlated with MMSE (β = −0.144, *p* = 5.44E−08), MOCA (β = −0.089, *p* = 4.07E−05), ADNI_EF (β = −0.073, *p* = 9.02E−06), ADNI_Mem (β = −0.09, *p* = 1.65E−09), ADNI_Lan (β = −0.082, *p* = 4.67E−05), and hippocampal volume (β = −0.028, *p* = 2.06E−06), and suggestively associated with ADNI_VS (β = −0.059, *p* = 0.01) and WMH (β = 0.02, *p* = 0.01) volume. Furthermore, plasma p-tau181 was suggestively associated with a longitudinal change of ADNI_VS (β = −0.05, *p* = 0.03) in the stage of MCI– and hippocampal volume (β = −0.03, *p* = 0.017) in the stage of pre-clinical AD. Furthermore, the plasma p-tau181 was significantly associated with ADNI_Mem (β = −0.17, *p* = 0.002) in the stage of AD dementia (Table 2). As demonstrated in Figure 4, plasma p-tau181 was correlated with annual rates of change in MMSE (β = −0.06, *p* < 2.0E−16), ADNI_EF (β = −0.02, *p* = 1.26E−10), ADNI_Mem (β = −0.03, *p* < 2.0E−16), ADNI_Lan (β = −0.02, *p* = 4.49E−12), ADNI_VS (β = −0.007, *p* = 4.48E−05), and hippocampal volume (β = −0.01, *p* = 0.0005).

**Table 2:** Baseline plasma p-tau 181 and change in volume of hippocampus and WMH and cognition.

		**CU–**		**MCI–**		**Preclinical AD**		**Prodromal AD**		**Dementia**	
		**β**	***p*-value**	**β**	***p*-value**	**β**	***p*-value**	**β**	***p*-value**	**β**	***p*-value**
MMSE	P-tau 181	0.010	0.869	0.025	0.674	0.006	0.954	−0.024	0.468	0.002	0.976
	time	−0.047	0.139	−0.030	0.200	−0.075	0.160	−0.261	<2E−16	−0.619	2.62E−11
	P-tau 181*time	0.006	0.841	0.003	0.912	−0.027	0.597	**−0.144**	**5.44E−08**	−0.099	0.219
MOCA	P-tau 181	0.031	0.650	−0.015	0.820	−0.073	0.481	−0.126	3.35E−03	−0.049	0.592
	time	−0.007	0.806	0.067	0.001	−0.036	0.404	−0.185	6.18E−16	−0.510	1.66E−12
	P-tau 181*time	−0.017	0.537	−0.009	0.632	−0.038	0.350	**−0.089**	**4.07E−05**	−0.076	0.178
ADNI_EF	P-tau 181	0.037	0.561	−0.099	0.121	−0.076	0.566	−0.114	0.021	−0.113	0.224
	time	0.006	0.771	0.025	0.067	−0.031	0.375	−0.101	2.28E−09	−0.339	3.89E−08
	P-tau 181*time	−0.019	0.336	−0.005	0.708	−0.030	0.372	**−0.073**	**9.02E−06**	−0.063	0.258
ADNI_Mem	P-tau 181	0.018	0.777	−0.065	0.328	−0.078	0.498	−0.207	6.50E−06	0.021	0.814
	time	0.029	0.122	0.047	0.007	−0.002	0.932	−0.146	<2E−16	−0.411	4.00E−11
	P-tau 181*time	−0.027	0.141	0.014	0.415	−0.023	0.354	**−0.090**	**1.65E−09**	**−0.174**	**0.002**
ADNI_Lan	P-tau 181	0.006	0.931	0.017	0.806	−0.241	0.046	−0.121	0.008	−0.169	0.074
	time	−0.006	0.791	0.008	0.626	−0.060	0.022	−0.134	8.09E−11	−0.386	1.19E−12
	P-tau 181*time	−0.023	0.299	−0.011	0.505	0	0.998	**−0.082**	**4.67E−05**	−0.043	0.356
ADNI_VS	P-tau 181	0.008	0.907	−0.144	0.030	0.153	0.210	−0.041	0.412	−0.003	0.978
	time	−0.020	0.529	−0.043	0.086	−0.023	0.629	−0.08	8.94E−04	−0.332	6.83E−07
	P-tau 181*time	−0.032	0.295	**−0.055**	**0.030**	−0.025	0.594	**−0.059**	**0.014**	−0.083	0.171
Hippocampal	P-tau 181	−0.034	0.619	−0.020	0.764	0.154	0.282	−0.034	0.541	0.009	0.925
volume	time	−0.075	<2E−16	−0.060	<2E−16	−0.117	5.42E−12	−0.123	<2E−16	−0.187	<2E−16
	P-tau 181*time	−0.011	0.106	−0.002	0.771	**−0.031**	**0.017**	**−0.028**	**2.06E−06**	−0.007	0.691
WMH	P-tau 181	6.13E−05	0.999	−0.131	0.035	0.078	0.606	0.063	0.288	0.123	0.183
volume	time	0.077	3.97E−05	0.041	1.38E−06	0.031	0.004	0.063	5.43E−13	0.064	0.026
	P-tau 181*time	−0.027	0.146	0.012	0.148	0.007	0.472	**0.020**	**0.010**	0.025	0.380

*CU–, cognitively unimpaired with negative Aβ; MCI–, mild cognitive impairment with negative Aβ; MMSE, mini-mental state examination; MOCA, Montreal cognitive assessment; ADNI_EF, executive domain composite score; ADNI_Mem, memory domain composite score; ADNI_Lan, language-related composite score; ADNI_VS, visuospatial domain composite score. Significant longitudinal effects are bolded*.

### Analyses of Longitudinal Conversion to Dementia

**Figure 5:**
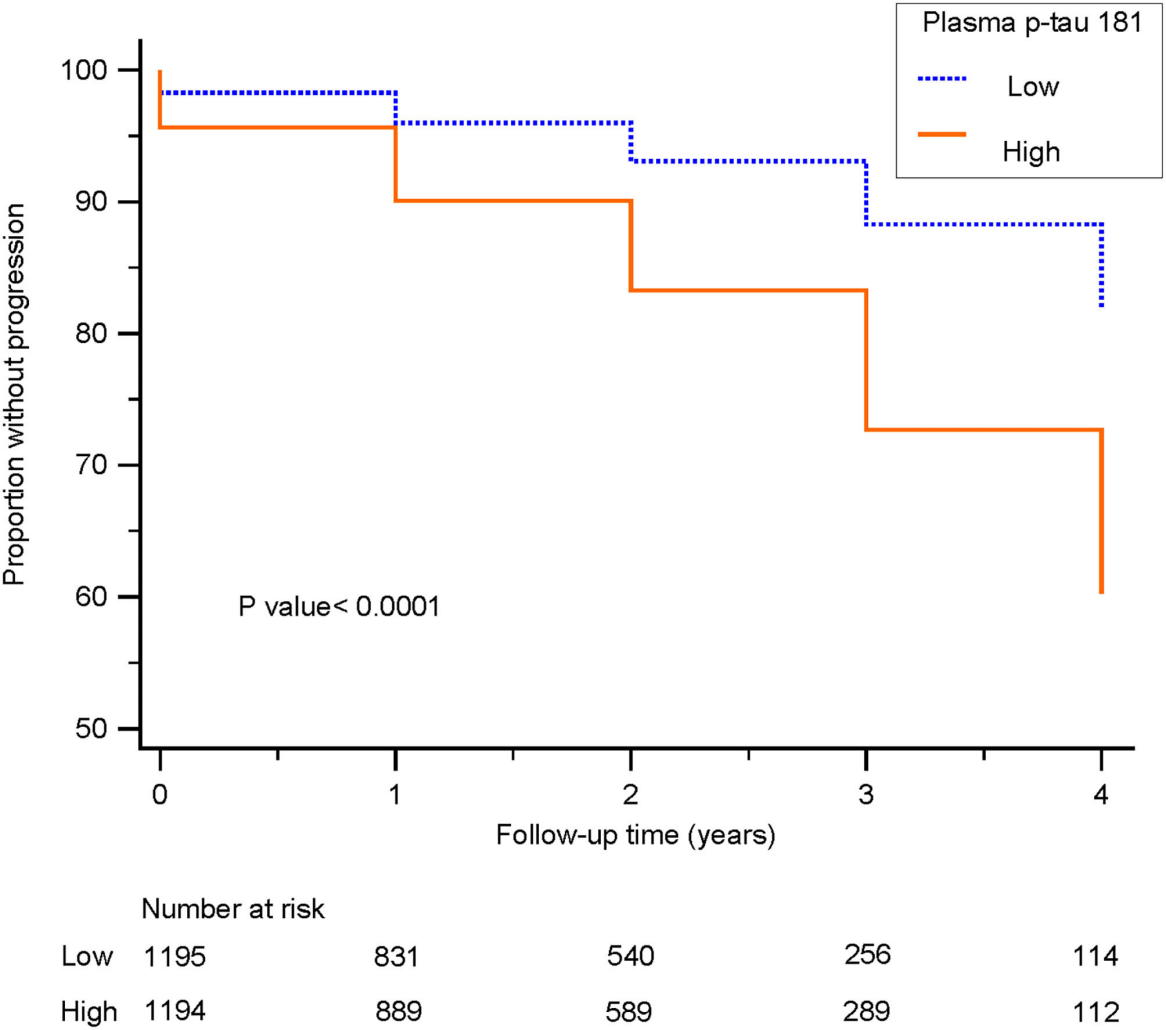
Survival curves for patients with low and high levels of plasma p-tau 181 are shown. Survival curves delineate subjects with cognitively unimpaired or mild cognitive impairment progressing to dementia. Blue line: low level, patients with plasma p-tau 181 levels <14.981 pg/ml; orange line: high level, patients with plasma p-tau 181 levels higher than 14.982 pg/ml.

**Figure 6:**
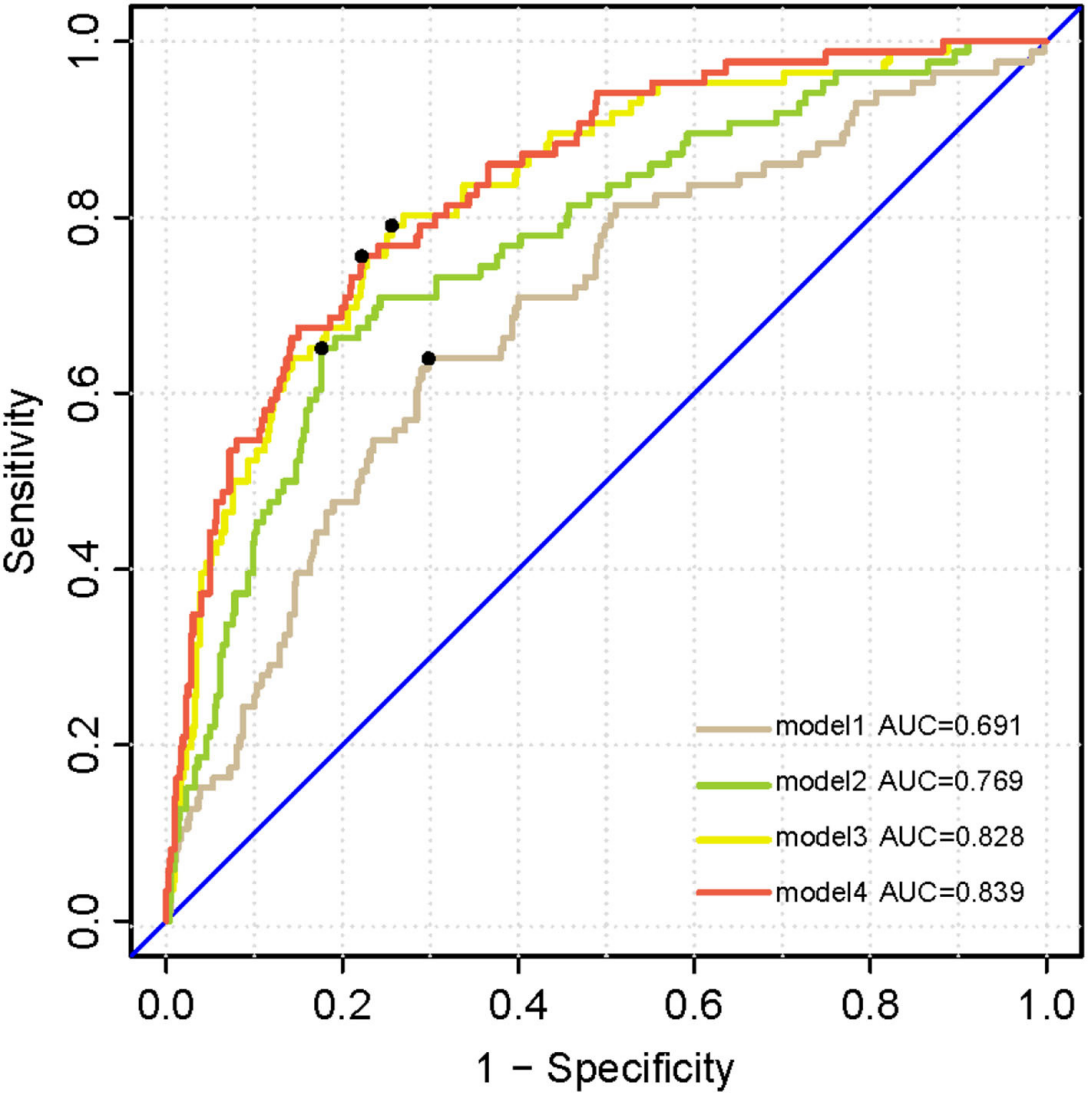
Receiver operating characteristic analysis curves denoting CSF biomarkers and plasma p-tau 181 for progressing to dementia. Model 1 includes age, sex, years of education, and APOE ε4 genotype. Model 2 additionally includes plasma p-tau 181. Model 3 additionally accounting for CSF biomarkers including Aβ1-42, t-tau, and p-tau. All above factors are contained in model 4.

Kaplan–Meier survival analysis demonstrated that subjects with higher levels (>14.982 pg/ml) of plasma p-tau181 were associated with higher progression compared to those with lower levels (<14.981 pg/ml) of plasma p-tau181 (log-rank *p* < 0.0001; Figure 5). The basic model (model 1: age, sex, education, and APOE ε4 genotype) discriminated clinical progression with the AUC of 0.69; when it included plasma, p-tau181 (model 2) was 0.77, Aβ1-42, t-tau, and p-tau of CSF (model 3) was 0.83, and all above (model 4) included was 0.84 (Figure 6).

## Discussion

This longitudinal study demonstrated that plasma p-tau181 level was elevated during the clinical progression of dementia, and associations with CSF biomarkers, different cognitive domains, and imaging markers were validated. Our findings highlighted that plasma p-tau181 was (1) positively associated with CSF t-tau and p-tau in pre-clinical AD, prodromal AD, and dementia stage; (2) longitudinally correlated with change and change rate of global cognition, executive function, memory, language-related, and visuospatial domains and hippocampal volume; (3) significantly predictive of clinical conversion to dementia.

We showed that plasma p-tau181 of subjects with positive Aβ were higher than subjects with negative Aβ. Consistent with previous investigations, increased plasma p-tau181 level was associated with increased Aβ pathology (24). Moreover, the change of plasma p-tau181 occurred before the onset of abnormal Aβ pathology, indicating that accumulation of Aβ could stimulate the early increase in plasma p-tau181 (25). As expected, strong associations were found between plasma and CSF levels of p-tau181, indicating that plasma p-tau181 might be used as a surrogate marker of tau pathology, as well as CSF p-tau (17, 18). However, the DELCODE study had demonstrated that plasma tau was not related to core markers of CSF in the stage of subjective cognitive decline (26). In line with the previous study (27), there was no association between plasma p-tau181 and CSF p-tau in subjects with negative Aβ. Inconsistently, we also found that plasma p-tau181 was associated with CSF Aβ in CU with negative Aβ, caused by the more specific subgroups (27). Additionally, plasma p-tau181 was significantly related to CSF t-tau/Aβ and p-tau/Aβ ratios, but not CSF Aβ, in pre-clinical AD and prodromal AD. This was supported by previous studies that ratios were more accurate biomarkers than single ones in predicting the progression of AD (28, 29).

Previous studies had demonstrated that plasma p-tau181 was correlated with cognitive decline (18, 30), and we found consistent associations between increased plasma p-tau181 and decreased cognitions in the whole cohort. Interestingly, the baseline plasma p-tau181 had an *S*-shaped relationship with global cognition and multiple cognitive domains in our study. Besides, in the stage of prodromal AD, the baseline plasma p-tau181 showed obvious correlations with changes of cognitions and hippocampal atrophy. The associations of plasma p-tau181 with hippocampal atrophy and gray matter loss indicated that plasma p-tau181 was related to Alzheimer's neuronal loss (30). Furthermore, the linear mixed-effects model analysis suggested that plasma p-tau181 related to WMH volume in prodromal AD (*p* = 0.01). From this, it could be speculated that plasma p-tau181 was relevant to cerebrovascular disease. What is more, associations are also presented as *S*-shaped patterns between plasma p-tau181 and change rates of global cognition, executive function, memory, language-related, and visuospatial abilities, as well as hippocampal atrophy rate. Therefore, it could be hypothesized that there is a threshold period for p-tau181 and only moderate levels of p-tau181 are associated with change rates of the above indicators. After a specific range of p-tau181, these effects are gradually leveling out.

The changes of phosphorylation sites are different at different stages of AD progression, besides plasma p-tau217 and p-tau181 are increased 20 years before the formation of tau aggregates (31–33). Consistent with prior research, plasma p-tau181 was a potential biomarker for diagnosing AD and predicting AD progression (19, 34, 35). Plasma p-tau181 was a specific marker in AD dementia, which can distinguish AD from frontotemporal dementia, vascular dementia, progressive supranuclear palsy, corticobasal basal syndrome, primary progressive aphasia, Parkinson's disease, and multiple system atrophy (17). In patients with familial AD, the level of plasma p-tau181 was comparable among APP and PSEN1 mutation carriers (20). Though plasma p-tau181 changed without a specific gene, it was associated with APOE ε4 (36). The association between tau and APOE ε4 was also independent of Aβ, primarily mediated by activated microglia (37).

Nevertheless, several limitations should be addressed in this study. Firstly, the ADNI study is mainly based on Caucasoid populations, and consequently, the results may not be directly applied to other racial/ethnic groups. Secondly, due to the strict inclusion criteria, individuals with severe vascular pathology were excluded, which probably attenuated relevance between plasma p-tau181 and cerebrovascular diseases. Also, follow-up data of biomarkers were not complete, and therefore, we cannot test whether plasma p-tau181 was congruent with CSF p-tau181 and tau PET.

In summary, plasma p-tau181 was increased from the pre-clinical stage of AD and longitudinally associated with multiple cognitive domains decline and hippocampal atrophy. Thus, plasma p-tau181, a readily accessible biomarker, could be of high predictive and diagnostic values for AD. However, international multicenter, large-sample, and longitudinal studies are needed to validate these hypotheses.

## Data Availability Statement

The datasets presented in this study can be found in online repositories. The names of the repository/repositories and accession number(s) can be found in the article/Supplementary Material.

## Ethics Statement

The ADNI study was conducted according to the Good Clinical Practice guidelines, the Declaration of Helsinki, and US 21 CFR: Part 50 (Protection of Human Subjects) and Part 56 (Institutional Review Boards). The ADNI study was conducted in compliance with HIPAA regulations. The first author of this paper was granted administrative permissions to access the anonymized ADNI data in May, 2019. Ethics approval for data collection in ADNI was obtained by each ADNI participating institution's institutional review board (https://adni.loni.usc.edu/wp14%20content/uploads/how_to_apply/ADNI_Acknowledgement_List.pdf). All study participants or authorized representatives provided written informed consent. Ethics approval was obtained from the institutional review boards of each institution involved: Oregon Health and Science University; University of Southern California; University of California—San Diego; University of Michigan; Mayo Clinic, Rochester; Baylor College of Medicine; Columbia University Medical Center; Washington University, St. Louis; University of Alabama at Birmingham; Mount Sinai School of Medicine; Rush University Medical Center; Wien Center; Johns Hopkins University; New York University; Duke University Medical Center; University of Pennsylvania; University of Kentucky; University of Pittsburgh; University of Rochester Medical Center; University of California, Irvine; University of Texas Southwestern Medical School; Emory University; University of Kansas, Medical Center; University of California, Los Angeles; Mayo Clinic, Jacksonville; Indiana University; Yale University School of Medicine; McGill University, Montreal-Jewish General Hospital; Sunnybrook Health Sciences, Ontario; U.B.C.Clinic for AD & Related Disorders; Cognitive Neurology—St. Joseph's, Ontario; Cleveland Clinic Lou Ruvo Center for Brain Health; Northwestern University; Premiere Research Inst (Palm Beach Neurology); Georgetown University Medical Center; Brigham and Women's Hospital; Stanford University; Banner Sun Health Research Institute; Boston University; Howard University; Case Western Reserve University; University of California, Davis—Sacramento; Neurological Care of CNY; Parkwood Hospital; University of Wisconsin; University of California, Irvine—BIC; Banner Alzheimer's Institute; Dent Neurologic Institute; Ohio State University; Albany Medical College; Hartford Hospital, Olin Neuropsychiatry Research Center; Dartmouth-Hitchcock Medical Center; Wake Forest University Health Sciences; Rhode Island Hospital; Butler Hospital; UC San Francisco; Medical University South Carolina; St. Joseph's Health Care Nathan Kline Institute; University of Iowa College of Medicine; Cornell University; and University of South Florida: USF Health Byrd Alzheimer's Institute.

## Author Contributions

JX and Y-LW conceived and designed the study. Y-LW, JC, and Z-LD conducted, analyzed, and extracted data. Y-LW, JC, and HW contributed to the generation of the manuscript. F-ZW helped revise the manuscript. All authors contributed to the editing of the manuscript.

## Funding

This study was supported by the National Natural Science Foundation (Grant Numbers 82071187, 81870821, and 81471215) and the Beijing Youth Talent Team Support Program (2018000021223TD08).

## Conflict of Interest

HW was employed by company WeGene, Shenzhen Zaozhidao Technology Co. Ltd. The remaining authors declare that the research was conducted in the absence of any commercial or financial relationships that could be construed as a potential conflict of interest.

## Publisher's Note

All claims expressed in this article are solely those of the authors and do not necessarily represent those of their affiliated organizations, or those of the publisher, the editors and the reviewers. Any product that may be evaluated in this article, or claim that may be made by its manufacturer, is not guaranteed or endorsed by the publisher.
