# Supplementary material for: Plasma p-tau181 Level Predicts Neurodegeneration and Progression to Alzheimer's Dementia: A Longitudinal Study
Source: Front Neurol. 2021 Sep 7;12:695696. doi: 10.3389/fneur.2021.695696 (PMC8452983; doi:10.3389/fneur.2021.695696)
Supplement: Supplementary file 1 [file Table_1.DOCX]

Etable1. Cross-sectional associations of plasma p-tau 181 with CSF biomarkers across AD staging

|  | CU- | | MCI- | | CU+ | | MCI+ | | Dementia | | Total | |
| --- | --- | --- | --- | --- | --- | --- | --- | --- | --- | --- | --- | --- |
|  |  |  |  |  | (preclinical AD) | | (prodromal AD) | |  |  |  |  |
|  | β | P | β | P | β | P | β | P | β | P | β | P |
| CSF Aβ | -0.17 | 0.037* | 0.049 | 0.557 | -0.168 | 0.276 | -0.109 | 0.083 | -0.088 | 0.424 | -0.127 | 0.0003* |
| CSF T-tau | 0.054 | 0.511 | 0.049 | 0.542 | 0.462 | 0.001* | 0.231 | 0.0003* | 0.22 | 0.037* | 0.222 | 2.44E-09* |
| CSF P-tau | 0.063 | 0.436 | 0.064 | 0.428 | 0.512 | 0.0003* | 0.265 | 3.29E-05* | 0.254 | 0.016* | 0.252 | 5.70E-12* |
| CSF T-tau/Aβ | 0.247 | 0.0009* | -0.009 | 0.904 | 0.434 | 0.003* | 0.206 | 0.0008* | 0.153 | 0.144 | 0.21 | 5.87E-11* |
| CSF P-tau/Aβ | 0.242 | 0.001* | 0.008 | 0.917 | 0.451 | 0.002* | 0.232 | 0.0002* | 0.174 | 0.096 | 0.227 | 2.62E-12* |

Abbreviations: CU-, cognitively unimpaired with negative Aβ; MCI, mild cognitive impairment; Aβ, amyloid-β; T-tau, total tau protein; P-tau, phosphorylated tau protein
