# Supplementary material for: Plasma p-tau181 Level Predicts Neurodegeneration and Progression to Alzheimer's Dementia: A Longitudinal Study
Source: Front Neurol. 2021 Sep 7;12:695696. doi: 10.3389/fneur.2021.695696 (PMC8452983; doi:10.3389/fneur.2021.695696)
Supplement: Supplementary file 2 [file Table_2.DOCX]

Etable 2. Cross-sectional associations of plasma p-tau 181 with cognition and neuroimaging biomarkers

|  | Low | | Medium | | High | | Total | |
| --- | --- | --- | --- | --- | --- | --- | --- | --- |
|  | β | P | β | P | β | P | β | P |
| MMSE | -0.041 | 0.502 | -0.220 | 0.0003* | -0.020 | 0.740 | -0.174 | 1.18E-06* |
| MOCA | -0.028 | 0.646 | -0.171 | 0.005* | -0.038 | 0.523 | -0.228 | 2.87E-10* |
| ADNI_EF | 0.029 | 0.621 | -0.120 | 0.038* | -0.064 | 0.282 | -0.168 | 1.26E-06* |
| ADNI_Mem | -0.076 | 0.169 | -0.174 | 0.003* | -0.063 | 0.276 | -0.243 | 5.16E-13* |
| ADNI_Lan | 0.046 | 0.448 | -0.111 | 0.065 | 0.002 | 0.974 | -0.193 | 5.35E-08* |
| ADNI_VS | -0.061 | 0.323 | -0.178 | 0.005* | -0.095 | 0.123 | -0.120 | 0.002* |
| Hippocampal volume | -0.015 | 0.801 | -0.086 | 0.111 | 0.028 | 0.637 | -0.096 | 0.006* |
| WMH volume | 0.035 | 0.548 | 0.062 | 0.302 | 0.067 | 0.271 | 0.043 | 0.240 |
