# Supplementary figures and images for: Plasma p-tau181 Level Predicts Neurodegeneration and Progression to Alzheimer's Dementia: A Longitudinal Study
Source: Front Neurol. 2021 Sep 7;12:695696. doi: 10.3389/fneur.2021.695696 (PMC8452983; doi:10.3389/fneur.2021.695696)

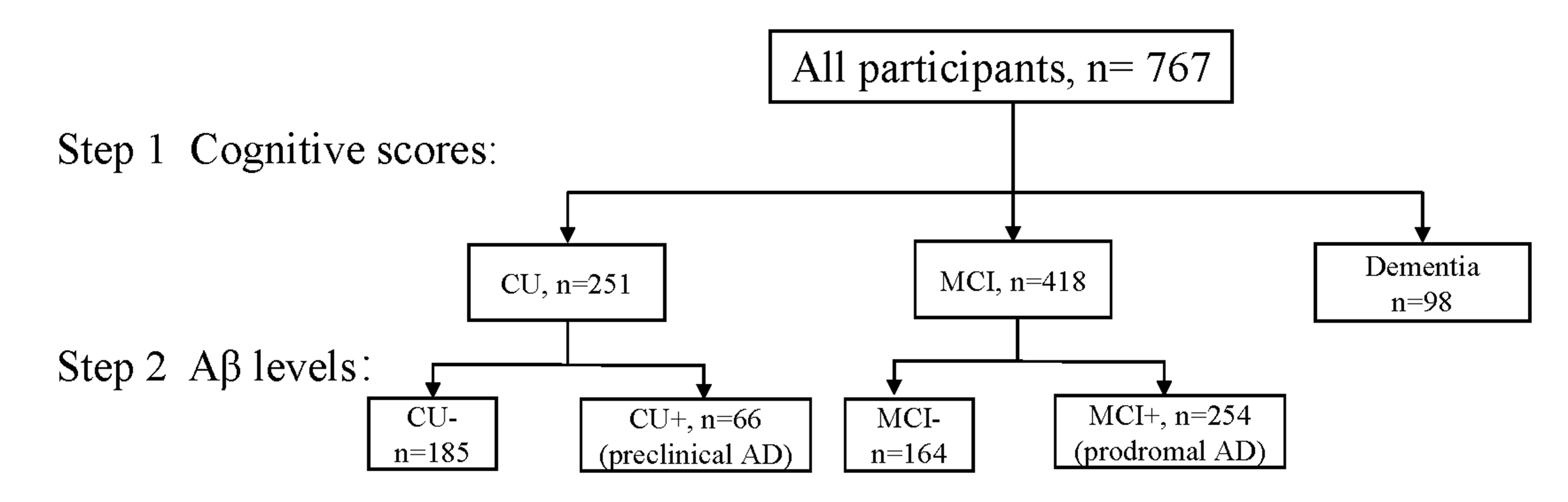

Supplement: Supplementary file 3 [file Image_1.tiff]
